# A streamlined workflow for long-read DNA methylation analysis with NanoMethViz and Bioconductor

**DOI:** 10.12688/f1000research.155204.1

**Published:** 2024-10-17

**Authors:** Shian Su, Lucinda Xiao, James Lancaster, Tamara Cameron, Kelsey Breslin, Peter F. Hickey, Marnie E. Blewitt, Quentin Gouil, Matthew E. Ritchie

**Affiliations:** 1 Walter and Eliza Hall Institute of Medical Research, Parkville, Victoria, Australia

**Keywords:** DNA methylation, Differential methylation, Long-read sequencing, Epigenetics, Bioconductor

## Abstract

Long-read sequencing technologies have transformed the field of epigenetics by enabling direct, single-base resolution detection of DNA modifications, such as methylation. This produces novel opportunities for studying the role of DNA methylation in gene regulation, imprinting, and disease. However, the unique characteristics of long-read data, including the modBAM format and extended read lengths, necessitate the development of specialised software tools for effective analysis. The NanoMethViz package provides a suite of tools for loading in long-read methylation data, visualising data at various data resolutions. It can convert the data for use with other Bioconductor software such as bsseq, DSS, dmrseq and edgeR to discover differentially methylated regions (DMRs).

In this workflow article, we demonstrate the process of converting modBAM files into formats suitable for comprehensive downstream analysis. We leverage NanoMethViz to conduct an exploratory analysis, visually summarizing differences between samples, examining aggregate methylation profiles across gene and CpG islands, and investigating methylation patterns within specific regions at the single-read level. Additionally, we illustrate the use of dmrseq for identifying DMRs and show how to integrate these findings into gene-level visualization plots. Our analysis is applied to a triplicate dataset of haplotyped long-read methylation data from mouse neural stem cells, allowing us to visualize and compare the characteristics of the parental alleles on chromosome 7. By applying DMR analysis, we recover DMRs associated with known imprinted genes and visualise the methylation patterns of these genes summarised at single-read resolution. Through DMR analysis, we identify DMRs associated with known imprinted genes and visualize their methylation patterns at single-read resolution. This streamlined workflow is adaptable to common experimental designs and offers flexibility in the choice of upstream data sources and downstream statistical analysis tools.

## 1. Introduction

DNA methylation is a key epigenetic regulator of gene expression in mammals, involving the addition of a methyl group to cytosine or adenine nucleotides. In mammals, the predominant form of DNA methylation is 5-methylcytosine (5mC) in CpG dinucleotides. CpG methylation is involved in many important epigenetic processes, including differential gene expression, X-chromosome inactivation, genomic imprinting, and maintenance of genome stability. ^1^ Changes in CpG methylation are implicated in a variety of different disease processes, including tumorigenesis and imprinting disorders such as Prader-Willi Syndrome and Angelman Syndrome. ^2^

Over the past couple of decades, multiple methods have been developed to measure DNA methylation, including bisulfite sequencing, ^3^ methylation arrays, ^4^ and more recently, third-generation sequencing technologies such as those offered by Oxford Nanopore Technologies (ONT) and Pacific Biosciences (PacBio). While bisulfite sequencing has long been the gold standard for DNA methylation analysis, it suffers from several limitations; these include DNA degradation from the process of bisulfite conversion, incomplete conversion of unmethylated cytosines, amplification bias from PCR, and short read lengths that pose difficulties for assembly and haplotyping. ^5^ By contrast, ONT’s nanopore sequencing and PacBio’s single-molecule real-time (SMRT) sequencing can directly sequence DNA molecules with all their modifications intact, and use basecalling algorithms to predict DNA modifications from the raw sequencing signal. Basecalling algorithms have the potential for bias, depending on the algorithm and training data used for the model; however, improvements are continually being made to generate more accurate models, and additionally, to detect other DNA modifications in other sequence contexts. ^6^

In this workflow, we will demonstrate how to use the NanoMethViz package ^7^ and other Bioconductor software to visualise and analyse methylation data generated by ONT long-read sequencing. The sequencing data is currently output in the modBAM file format, which contains the basecalled reads alongside tags for modification information. The NanoMethViz package provides a suite of tools for loading in this methylation data, visualising regional and genome-wide methylation patterns, and exporting data for identifying differentially methylated regions (DMRs) using other Bioconductor software such as bsseq, ^8^ DSS, ^9^ dmrseq ^10^ and edgeR ^11^.

## 2. Methods

### 2.1 Experimental setup

**Figure 1.**
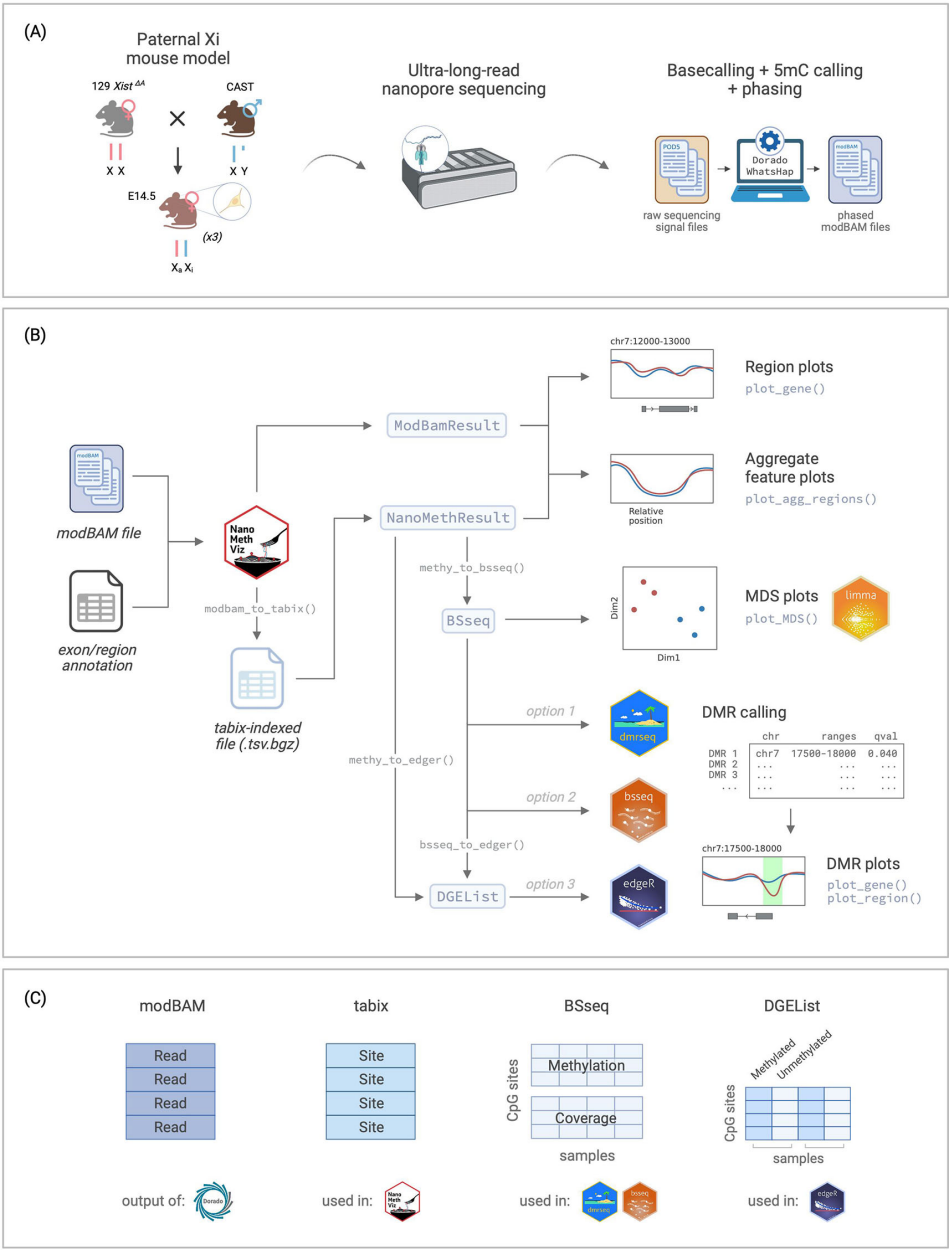
Overview of the workflow. (A) Experimental setup for the data used in this workflow, from Gocuk and Lancaster et al., 2024. (B) Flowchart of the workflow for long-read methylation data visualisation and analysis using NanoMethViz and other Bioconductor packages including limma, dmrseq, bsseq, and edgeR. (C) Summary of data structures for the storage of methylation data used in different parts of the workflow. Xi: inactive X. Xa: active X. CAST: Castaneus. 5mC: 5-methylcytosine. MDS: multidimensional scaling. DMR: differentially methylated region. Figure created with BioRender.com.

In this workflow, we will analyse ultra-long-read Nanopore sequencing data from the neural stem cells of triplicate E14.5 female mice from Gocuk and Lancaster *et al.,* 2024. ^12^ The mice were F1 crosses between an Xist knockout (X ^ΔA^X) female ^13^ and a Castaneus strain (CAST) male. The resulting offspring have sufficiently different parental chromosomes to allow effective genome-wide haplotyping to distinguish the parent-of-origin for long-reads, allowing for the identification of genes that are imprinted or strain-specific. The Xist KO of the maternal genome also guarantees that X-inactivation takes place on the paternal X chromosome, allowing for the study of X-inactivation. The DNA was sequenced on R9.4.1 PromethION flow cells, basecalled with Dorado, aligned to the mm10 mouse reference genome, and phased with WhatsHap ^14^ to produce the phased modBAM ( Figure 1A). For illustrative purposes, we use a subset of the data focusing on chromosome 7, which contains several known imprinted genes.

### 2.2 Loading software and data

We begin by loading the packages and downloading the input data which contains the modBAM files required for this workflow.

**library**(dmrseq)
**library**(dplyr)
**library**(readr)
**library**(plyranges)
**library**(NanoMethViz)
**set.seed**(1234)

**if** (**!dir.exists**("input")) {
    **options**(timeout = 600)
    **download.file**("https://zenodo.org/records/12747551/files/input.tar.gz?download=1", "input.tar.gz")
    utils**::untar**("input.tar.gz")
    **file.remove**("input.tar.gz")
}


As mentioned, modBAM files are the currently preferred output of Dorado, the primary methylation caller offered by ONT. ModBAM files are BAM files containing reads with additional tags on each read storing information about methylation calls (the MM tag) alongside their methylation probabilities (the ML tag). To use this data in NanoMethViz, we must construct an object of the ModBamResults class.

The ModBamResults class is a container for storing methylation data from multiple samples, along with sample annotation and exon annotation. To create this object, we need to provide paths to the modBAM files, a sample table, and optionally exon annotation.

*# List the modBAM files by searching for files with the .bam extension in the input directory*bam_files <- **dir**("input", pattern = "*bam$", full.names = TRUE)
bam_files

## [1] "input/nsc_1_mat.bam" "input/nsc_1_pat.bam" "input/nsc_2_mat.bam"
## [4] "input/nsc_2_pat.bam" "input/nsc_3_mat.bam" "input/nsc_3_pat.bam"


The samples need to be annotated with information for NanoMethViz to use in aggregating data within experimental groups. The sample table must contain sample and group columns with optional additional columns for further annotation. The rows of this table must match the order of the input files. The group column is generally the default grouping column used in the NanoMethViz package. In this example, we have grouped our data by haplotype.

samples <- **read_tsv**("input/sample_anno.tsv", show_col_types = FALSE)
samples

## # A  tibble: 6 × 4
##   sample     group   tissue  haplotype
##   <chr>      <chr>   <chr>   <chr>
## 1 nsc_1_mat  nsc_mat nsc     mat
## 2 nsc_1_pat  nsc_pat nsc     pat
## 3 nsc_2_mat  nsc_mat nsc     mat
## 4 nsc_2_pat  nsc_pat nsc     pat
## 5 nsc_3_mat  nsc_mat nsc     mat
## 6 nsc_3_pat  nsc_pat nsc     pat


Optionally we can add exon annotation to the object to generate tracks for gene annotation when plotting genomic regions/genes. The exon annotation must be a data.frame with columns gene_id, chr, strand, start, end, transcript_id, and symbol. Several helper functions are provided to retrieve exon annotations in the correct format for human (hg19, hg38) and mouse (mm10, GRCm39). In this example, we will use the get_exons_mm10() function to retrieve exon annotations for the mouse genome (mm10) and filter for chromosome 7.

exon_anno <- **get_exons_mm10**() **%>%**
    dplyr**::filter**(chr **==** "chr7")
exon_anno

## # A tibble:  60,146 × 7
##   gene_id    chr   strand   start      end transcript_id symbol
##   <chr>      <chr> <chr>    <int>    <int>         <int> <chr>
## 1 100009609  chr7  -     84935565 84941088         60883 Vmn2r65
## 2 100009609  chr7  -     84943141 84943264         60883 Vmn2r65
## 3 100009609  chr7  -     84943504 84943722         60883 Vmn2r65
## 4 100009609  chr7  -     84946200 84947000         60883 Vmn2r65
## 5 100009609  chr7  -     84947372 84947651         60883 Vmn2r65
## 6 100009609  chr7  -     84963816 84964115         60883 Vmn2r65
## 7 100009609  chr7  -     84935565 84941088         60884 Vmn2r65
## 8 100009609  chr7  -     84943141 84943264         60884 Vmn2r65
## 9 100009609  chr7  -     84943504 84943722         60884 Vmn2r65
## 10 100009609 chr7  -     84946200 84947000         60884 Vmn2r65
## # 60,136 more rows


**Figure 2.**
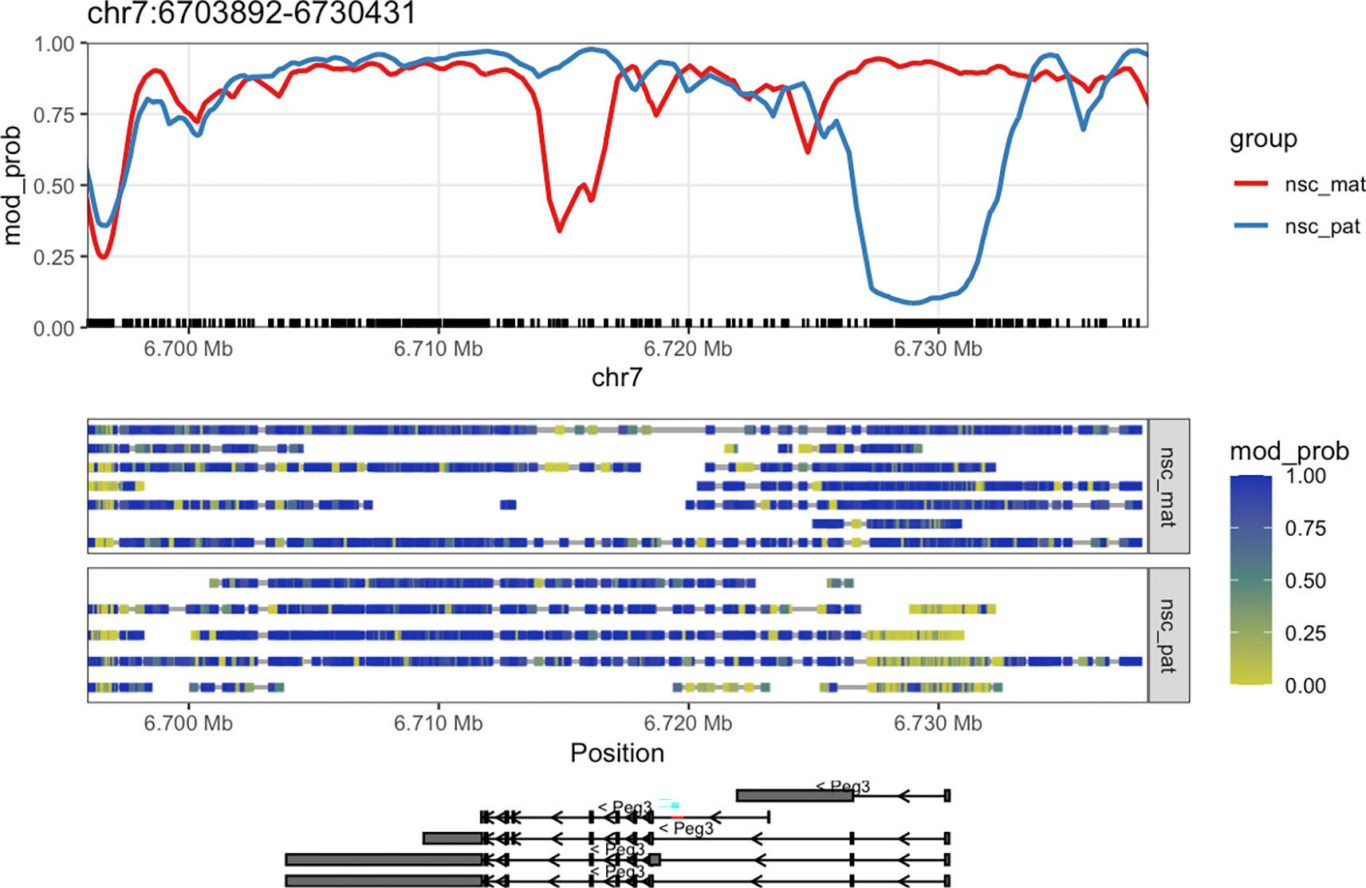
Gene-level plot of *Peg3.* This plot shows the methylation information along the *Peg3* gene on chromosome 7. The top track shows a rolling-smoothed average of methylation probability across the region, aggregated across samples within each experimental group. The middle track shows a heatmap of the methylation probabilities within individual reads, separated by experimental group. The bottom track shows isoform annotations for *Peg3*, with rectangles representing exons, lines representing introns and arrows representing the direction of transcription.

Once we have created the ModBamResult object, we can use the plot_gene() function to visualise the methylation patterns of a gene of interest. In this example, we will plot the methylation patterns of the *Peg3* (paternally-expressed gene 3) gene on chromosome 7, which is known to be imprinted in mice. The plot contains a smoothed trendline of the group-aggregated methylation probabilities across the gene, along with a heatmap showing the methylation probabilities of individual CpG sites in each read, and a track showing the isoforms of the gene if exon annotation is provided ( Figure 2).

mbr <- **ModBamResult**(
    methy = **ModBamFiles**(
        paths = bam_files,
        samples = samples**$**sample
   ),
   samples = samples,
   exons = exon_anno,
   mod_code = "m"
)

**plot_gene**(mbr, "Peg3")


CpG dinucleotides are often enriched around gene promoters, where methylation is generally correlated with silencing and hypomethylation with activation. In the plot, we see that there is a strong demethylation pattern on the paternal allele near the transcription start site (TSS) of the gene, allowing the gene to be expressed. The maternal allele is hypermethylated near the TSS, silencing the gene. This is a typical methylation pattern for an imprinted gene, and consistent with paternal expression of *Peg3.*

### 2.3 Genome-wide analysis

While it is possible to use modBAM files to analyse specific regions of interest, genome-wide analysis requires the data to be in a tabix-indexed TSV file as it is easier to parse at scale (Figure C). We can convert the modBAM files to a tabix-indexed TSV file using the modbam_to_tabix() function. This function will create a tabix-indexed TSV file containing the methylation data from the modBAM files. This file can then be used as input to the NanoMethViz functions that require sorted genomic data. In this example, we will convert the ModBamResult object to a tabix-indexed TSV file and save it to the data directory. We will use a pre-generated file in the interest of time, but you can run the code below to generate the file.

**dir.create**("data", showWarnings = FALSE)
**if** (**!file.exists**("data/methy.tsv.bgz")) {
    **modbam_to_tabix**(mbr, "data/methy.tsv.bgz")
}


Once we have the tabix-indexed TSV file, we can create a NanoMethResult object using the NanoMethResult() function. The NanoMethResult object contains the same information as the ModBamResult object but with the methylation data stored in a tabix-indexed TSV file. To create this object we need to provide paths to the tabix-indexed TSV file, along with the accompanying sample table and exon annotation we generated before.

nmr <- **NanoMethResult**(
    methy = "data/methy.tsv.bgz",
    samples = samples,
    exons = exon_anno
)


The NanoMethResult object behaves in the same way as ModBamResult for any plotting functions. For example we can use plot_gene() to visualise the methylation patterns of the *Peg3* gene in exactly the same way but replacing the ModBamResult object with the NanoMethResult object. We should see the exact same plot as before.

**plot_gene**(nmr, "Peg3")


### 2.4 Aggregating feature methylation data

It is often informative to aggregate methylation data over a class of features to identify broad patterns of methylation across the features. This can help establish overall differences in methylation patterns between groups in a class of features such as genes, CpG islands, or enhancers. The NanoMethViz package provides a plot_agg_regions() function for visualising methylation data that has been aggregated across a set of genomic regions of interest, defined by a table of coordinates.

**Figure 3.**
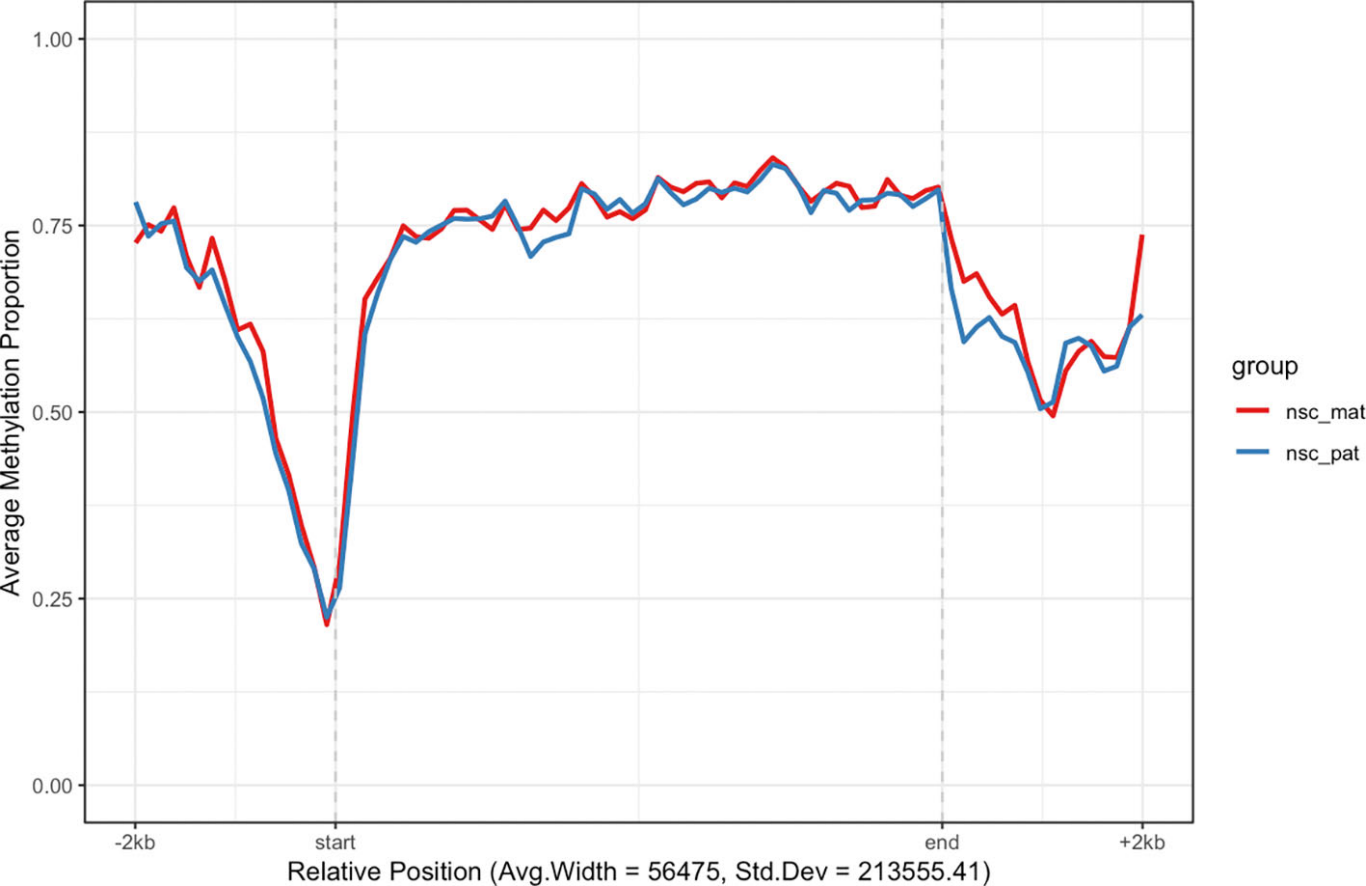
Aggregated gene-level plot. The mean methylation proportion is shown across a sample of 200 genes in chromosome 7, aggregated by experimental group. Methylation proportions are individual CpG sites are calculated along relative genomic positions, with fixed flanking regions, then averaged across all genes in each experimental group to produce a smoothed trendline. The x-axis label gives the average width and standard deviation of widths of the features used.

For example, we may want to investigate the methylation patterns of a set of genes on chromosome 7. We can use the exons_to_genes() helper function to convert exon annotations already stored in the object to gene annotations and filter for only chromosome 7 genes ( Figure 3). For faster processing, we subset 200 genes and use the plot_agg_regions() function to visualise the mean methylation proportions along the sampled genes. By default, the methylation proportion is calculated using a threshold of 0.5 modification probability.

gene_anno <- **exons_to_genes**(**exons**(nmr))
**plot_agg_regions**(nmr, regions = **slice_sample**(gene_anno, n = 200), group_col = "group")


In this plot we can see that active genes on chromosome 7 tend to be demethylated near the TSS region and hypermethylated throughout the gene body. This is consistent with the proposed function of methylation in gene regulation, where methylation in promoter region of genes is associated with gene silencing, while transcription of active genes is associated with recruitment of methylating mechanisms to the gene body.

**Figure 4.**
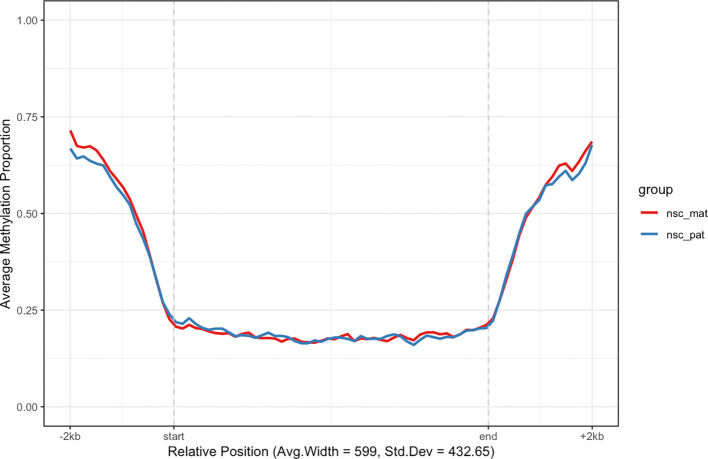
Aggregated CpG island-level plot. The mean methylation proportion is shown across CpG islands on chromosome 7, aggregated by experimental group. Methylation proportions are individual CpG sites are calculated along relative genomic positions, with fixed flanking regions, then averaged across all CpG islands in each experimental group to produce a smoothed trendline.

Another class of features of interest are CpG islands, which are regions of the genome that are rich in CpG dinucleotides. CpG islands are often associated with gene promoters and are predominantly unmethylated in normal cells. ^15^ We can use the get_cgi_mm10() function to retrieve CpG island annotations for the mouse genome (mm10) and filter for chromosome 7. We can then use the plot_agg_regions() function to visualise the mean methylation profile across CpG islands on chromosome 7 ( Figure 4).

cgi_anno <- **get_cgi_mm10**() **%>%**
   **filter**(chr **==** "chr7")
cgi_anno

## # A tibble: 1,193 × 14
##       bin   chr   start     end  gene_id length cpgNum gcNum perCpg perGc obsExp
##    <dbl> <chr>   <dbl>   <dbl>    <chr> <dbl>  <dbl> <dbl>  <dbl> <dbl> <dbl>
##  1   609  chr7 3181031 3181267  CpG: 22   236     22   147   18.6  62.3     1
##  2   610  chr7 3303442 3303817  CpG: 28   375     28   249   14.9  66.4  0.69
##  3   610  chr7 3366695 3366942  CpG: 27   247     27   152   21.9  61.5  1.22
##  4   611  chr7 3411983 3412269  CpG: 26   286     26   188   18.2  65.7  0.84
##  5   611  chr7 3414874 3415855  CpG: 87   981     87   518   17.7  52.8  1.28
##  6   611  chr7 3423522 3423834  CpG: 28   312     28   215   17.9  68.9  0.87
##  7   612  chr7 3617298 3617534  CpG: 17   236     17   146   14.4  61.9  0.76
##  8   612  chr7 3629691 3630056  CpG: 32   365     32   195   17.5  53.4  1.24
##  9   612  chr7 3645002 3645963  CpG: 106  961    106   652   22.1  67.8  0.96
## 10   612  chr7 3665238 3665517  CpG: 27   279     27   184   19.4  65.9  0.93
## # 1,183 more rows
## # 3 more variables: transcript_id <chr>, strand <chr>, symbol <chr>

**plot_agg_regions**(nmr, cgi_anno, group_col = "group")


As expected, we see a clear pattern of demethylation over CpG islands on chromosome 7, relative to neighbouring genomic regions.

### 2.5 Differential methylation

NanoMethViz is primarily designed for visualising methylation data. In order to perform differential methylation analysis, we can use other Bioconductor packages that implement sophisticated statistical procedures, such as dmrseq. The dmrseq() function requires a BSseq object as input, so we convert the NanoMethResult object to a BSseq object using the methy_to_bsseq() function.

We remove any sites that are zero in every samples of either experimental group; these low coverage regions are generally not informative for differential methylation analysis. The BSseq object stores the methylation data as two matrices, the methylation matrix and the coverage matrix ( Figure 1C), with the option to store additional information such as sample annotation and genomic regions.

This conversion process usually takes some time, so we will load a pre-generated BSseq object. The code used to generate the object is provided below.

**if** (**!file.exists**("data/bss.rds")) {
    bss <- **methy_to_bsseq**(nmr)
    **saveRDS**(bss, "data/bss.rds")
} **else** {
    bss <- **readRDS**("data/bss.rds")
}

pat_cov <- **getCoverage**(bss)[, **which**(bss**$**group **==** "nsc_pat")]
mat_cov <- **getCoverage**(bss)[, **which**(bss**$**group **==** "nsc_mat")]
low_cov <- (**rowSums**(pat_cov **==** 0) **==** 3) **|** (**rowSums**(mat_cov **==** 0) **==** 3)

**proportions**(**table**(low_cov))

## low_cov
##     FALSE      TRUE
## 0.2853227 0.7146773

bss <- bss[**!**low_cov, ]


### 2.6 MDS plot

Using the BSseq object, we can create a multi-dimensional scaling (MDS) plot to visualise the relative differences in methylation between the samples, a method commonly used in differential gene expression analysis. The MDS function within NanoMethViz uses the plotMDS() function from limma. ^16^ Since BSseq objects store the methylation data as two matrices, the methylation matrix and the coverage matrix, we need to convert the BSseq object such that each CpG site from each sample is represented by a single value in a matrix. This can be done using the bsseq_to_log_methy_ratio() function, which converts the BSseq object to a matrix of the log of the methylation ratio with a small count added to prevent division by zero.

The plot_mds() function can then be used to create an MDS plot of the samples using the log methylation ratio matrix as input. The samples are coloured by group – in this case, maternal and paternal.

**Figure 5.**
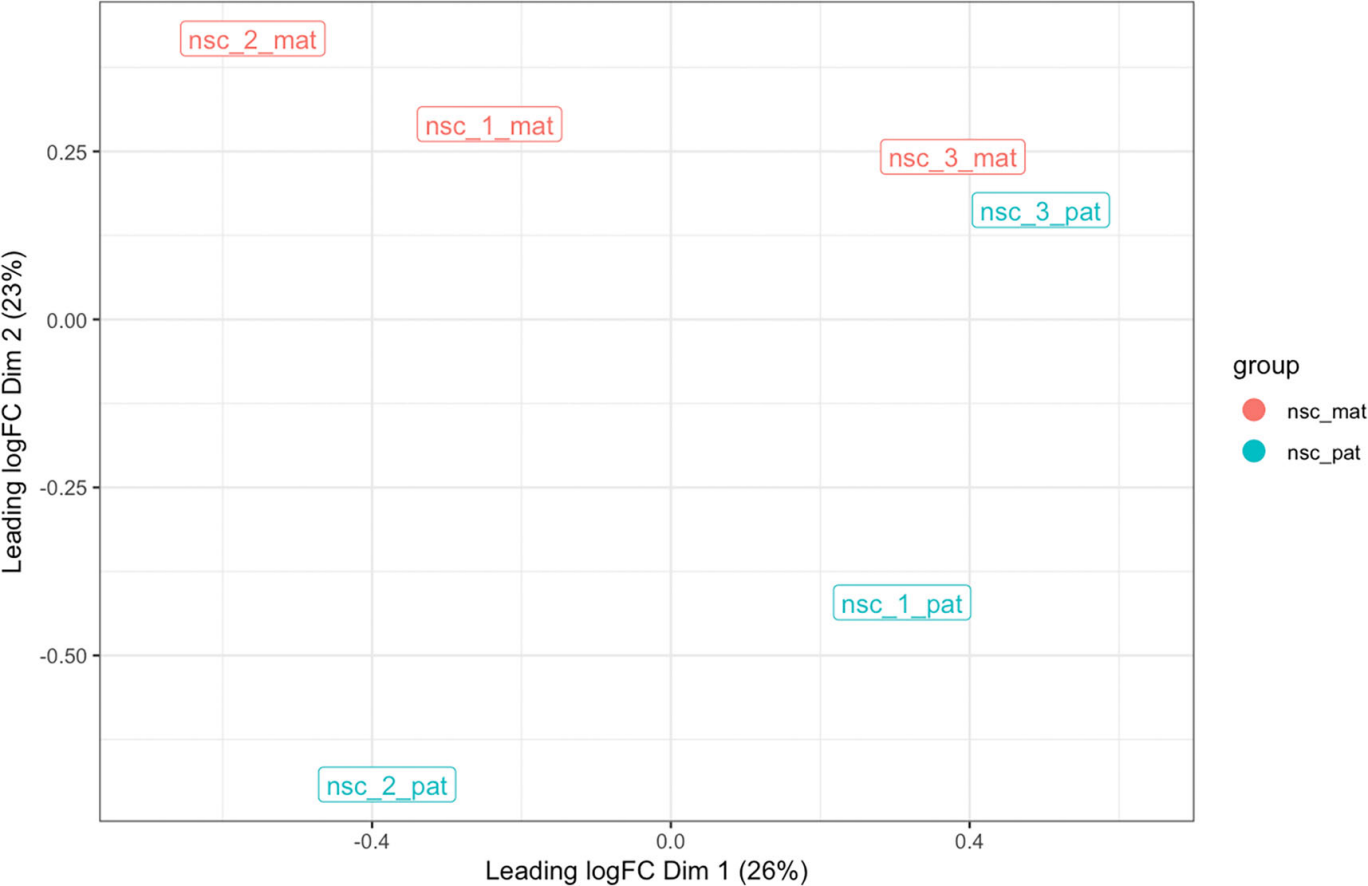
MDS plot. The multi-dimensional plot is created based on the top 500 most variable genes in on chromosome 7 in terms of log-methylation-ratio over the gene bodies. The samples are coloured by group, and distances between samples is reflective of the difference in the methylation profile across genes in each sample.

Because the number of CpG sites is large, here we aggregate the methylation data over CpG islands to both reduce the size of the data and produce a more stable measure of methylation. This is done by providing the regions = cgi_anno argument to bsseq_to_log_methy_ratio(). Without the argument, the function will use individual CpG sites. After the data is aggregated, plot_mds() uses only the top 500 most variable features to create the MDS plot ( Figure 5).

lmr <- **bsseq_to_log_methy_ratio**(
    bss,
    regions = cgi_anno
)

**plot_mds**(lmr, groups = bss**$**group)


From this MDS, we see that there is clear separation between the maternal and paternal groups based on CpG islands on chromosome 7, suggesting that the most variable CpG islands are differentially methylated between the two groups. Given that this is autosomal data, the separation is likely due to imprinting or strain-specific effects. We therefore expect to find some differentially methylated regions between the maternal and paternal groups.

### 2.7 Differentially methylated regions

Another common analysis is to identify differentially methylated regions (DMRs) between experimental groups. It is often informative to identify DMRs rather than just single differentially-methylated CpG sites, as these may correspond to gene regulatory regions such as promoters, silencers and enhancers, and have biological relevance for differential epigenetic regulation. However, DMRs are not easy to identify, as DMR calling requires the identification of individual significant sites, some determination of whether nearby sites should be aggregated into a region, and a statistical test to determine if the region as a whole is differentially methylated.

The dmrseq package can be used to identify DMRs between the two groups in our data. The dmrseq() function from the package requires a BSseq object as input, along with the name of the covariate to test for differential methylation. We will test for differential methylation between the maternal and paternal groups. The dmrseq() function returns a GRanges object containing the DMRs identified by the function. The GRanges object contains the genomic coordinates of the DMRs, along with information about the statistical significance of the DMRs.

There are alternatives to dmrseq for identifying DMRs, such as bsseq, DSS and edgeR. For bsseq and DSS, the same BSseq object can be used as input, while edgeR requires a DGEList object as input ( Figure 1C). The DGEList object can be created from the BSseq object using the bsseq_to_edger() function, or from the NanoMethResult object using the methy_to_edger() function ( Figure 1B).

bsseq, DSS and dmrseq all use a similar approach to identify DMRs: first, they use a statistical test to compare the methylation levels between the two groups at each CpG site, followed by a clustering step to identify regions of the genome with consistent differences in methylation levels for de novo DMR discovery. On the other hand, edgeR requires users to choose the regions to test for differential methylation, which is reflected by the regions over which they choose to summarise counts, and therefore cannot be used for de novo DMR discovery. dmrseq and edgeR produce p -values for each region, while bsseq and DSS only provide p -values for each CpG site along with an aggregated area statistic.

**if** (**!file.exists**("data/regions.rds")) {
    regions <- dmrseq**::dmrseq**(bss, testCovariate = "group", minNumRegion = 20)
    **saveRDS**(regions, "data/regions.rds")
} **else** {
    regions <- **readRDS**("data/regions.rds")
}


A number of regions will be produced by dmrseq, including regions that were aggregated because they contained significant CpG sites but subsequently failed to reach statistical significance at a region level. It is difficult to directly interpret the regions returned by differential methylation analysis, and we commonly wish to identify these regions are possible control regions for genes. We do this by associating DMRs with genes using proximity to the gene transcription start site (TSS), first by constructing an annotation of a promoter region, then overlapping it with the DMRs. We use a liberal window of 10kb around the TSS to capture potential promoter regions, this is done by transforming the gene annotation to a GRanges object, then using the promoters() function from IRanges ^17^ to define the promoter region as 5kb upstream and 5kb downstream of the TSS. We then use the join_overlap_intersect() function from plyranges to find the DMRs that overlap with the gene TSS regions. This will return a GRanges object containing the regions where DMRs which overlap with the promoter region, and the associated gene in that region.

gene_anno_gr <- **as**(gene_anno, "GRanges")
gene_anno_gr_tss <- IRanges**::promoters**(gene_anno_gr, upstream = 5000, downstream = 5000)

gene_dmr_overlaps <- plyranges**::join_overlap_intersect**(regions, gene_anno_gr_tss)
gene_dmr_overlaps

## GRanges object with 868 ranges and 9 metadata columns:
##       seqnames               ranges strand |        L        area        beta
##          <Rle>            <IRanges>  <Rle> | <integer>  <numeric>   <numeric>
##    [1]    chr7      6727308-6731838      * |      157    128.8218    -1.84082
##    [2]    chr7      6727308-6731838      * |      157    128.8218    -1.84082
##    [3]    chr7  143294268-143296757      * |      112     92.9051    -1.87215
##    [4]    chr7    60003279-60005228      * |       60     50.9278    -1.82583
##    [5]    chr7    60003279-60005228      * |       60     50.9278    -1.82583
##     …        …                    …      …          …           …           …
##  [864]    chr7  126579062-126580889      * |       27     5.00269   0.0232921
##  [865]    chr7  126580818-126580889      * |       27     5.00269   0.0232921
##  [866]    chr7  126579942-126580889      * |       27     5.00269   0.0232921
##  [867]    chr7    19696925-19697636      * |       23     2.65049  -0.0118438
##  [868]    chr7    19696925-19697636      * |       23     2.65049  -0.0118438
##              stat        pval      qval         index     gene_id      symbol
##         <numeric>   <numeric> <numeric>     <IRanges> <character> <character>
##    [1]   -40.6010 5.71657e-05 0.0401684   27111-27267       18616        Peg3
##    [2]   -40.6010 5.71657e-05 0.0401684   27111-27267       57775       Usp29
##    [3]   -32.5253 1.14331e-04 0.0401684 910828-910939       63830    Kcnq1ot1
##    [4]   -20.1018 1.14331e-04 0.0401684 335287-335346       52480      Snhg14
##    [5]   -20.1018 1.14331e-04 0.0401684 335287-335346       84704       Snurf
##     …           …           …         …             …           …           …
##  [864]  0.0925951    0.996341  0.997876 764165-764191   102465641     Mir7059
##  [865]  0.0925951    0.996341  0.997876 764165-764191       12752        Cln3
##  [866]  0.0925951    0.996341  0.997876 764165-764191      171504       Apobr
##  [867] -0.0795915    0.996456  0.997876   81437-81459       11812       Apoc1
##  [868] -0.0795915    0.996456  0.997876   81437-81459       11816        Apoe
## -------
## seqinfo: 1 sequence from an unspecified genome; no seqlengths


After we identify the DMRs that overlap with the gene TSS regions, we can filter for the significant DMRs using the qval column. This is the adjusted p -value for the DMRs, calculated using the Benjamini-Hochberg procedure to control false-discovery rates for multiple-testing. We can then select the columns we are interested in, such as the gene symbol, chromosome, start and end positions of the DMRs, the strand of the gene, and the q-value of the DMRs.

dmr_regions <- **as_tibble**(gene_dmr_overlaps) **%>%**
    dplyr**::rename**(chr = "seqnames")

signif_regions <- dmr_regions **%>%**
    dplyr**::filter**(qval **<** 0.05)

signif_regions **%>%**
    dplyr**::select**(symbol, chr, start, end, strand, qval)

## # A  tibble:   8 × 6
##   symbol     chr      start        end strand   qval
##   <chr>    <fct>      <int>      <int> <fct>   <dbl>
## 1 Peg3      chr7    6727308    6731838 *      0.0402
## 2 Usp29     chr7    6727308    6731838 *      0.0402
## 3 Kcnq1ot1  chr7  143294268  143296757 *      0.0402
## 4 Snhg14    chr7   60003279   60005228 *      0.0402
## 5 Snurf     chr7   60003279   60005228 *      0.0402
## 6 Mkrn3     chr7   62416953   62423032 *      0.0402
## 7 Cdkn1c    chr7  143459739  143462038 *      0.0402
## 8 Peg12     chr7   62461469   62464053 *      0.0402


**Figure 6.**
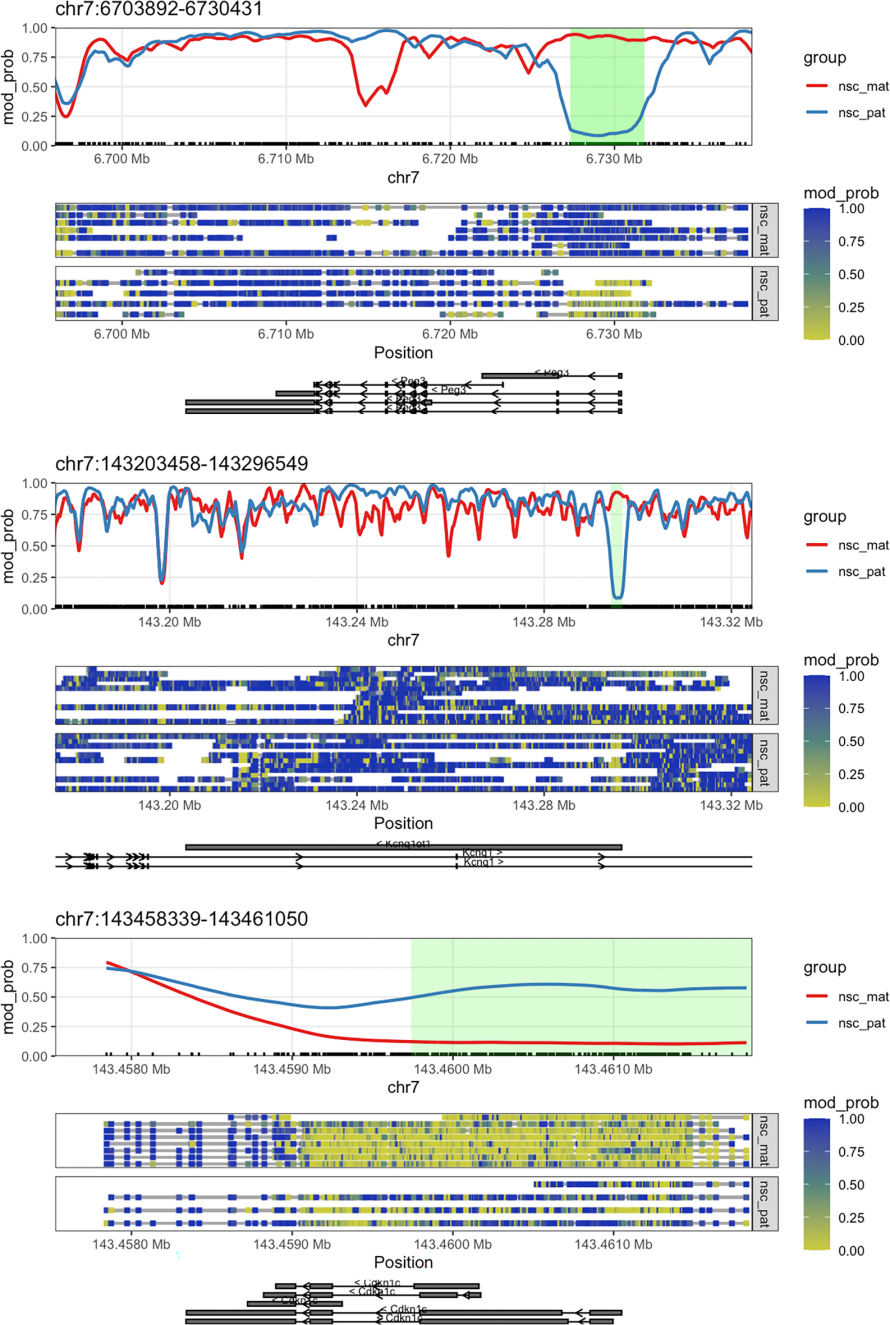
Gene plots with DMR. The plots show the genes *Peg3*, *Kcnq1ot1*, and *Cdkn1c* along with green highlighted region denoting the DMR discovered by dmrseq.

We now have a list of DMRs which overlap the TSS of genes on chromosome 7 and have a q-value < 0.05. The genes on this list are all known imprinted genes in the mouse, including *Peg3*, which we plotted in the first section of this workflow. We can plot the methylations of these genes and highlight the DMRs by setting the plot_gene() function’s anno_regions argument to the regions identified. This will highlight the significant regions in the gene plot in a shaded band. Here we will plot *Peg3*, *Kcnq1ot1*, and *Cdkn1c* ( Figure 6). We can see that for all three of these genes, as required by our filtering criteria, the differentially methylated regions fall near the TSS.

**options**("NanoMethViz.highlight_col" = "green")
(patchwork**::wrap_elements**(**plot_gene**(nmr, "Peg3", anno_regions = signif_regions)) **/** patchwork**::wrap_elements**(**plot_gene**(nmr, "Kcnq1ot1", anno_regions = signif_regions)) **/** patchwork**::wrap_elements**(**plot_gene**(nmr, "Cdkn1c", anno_regions = signif_regions)))


**Figure 7.**
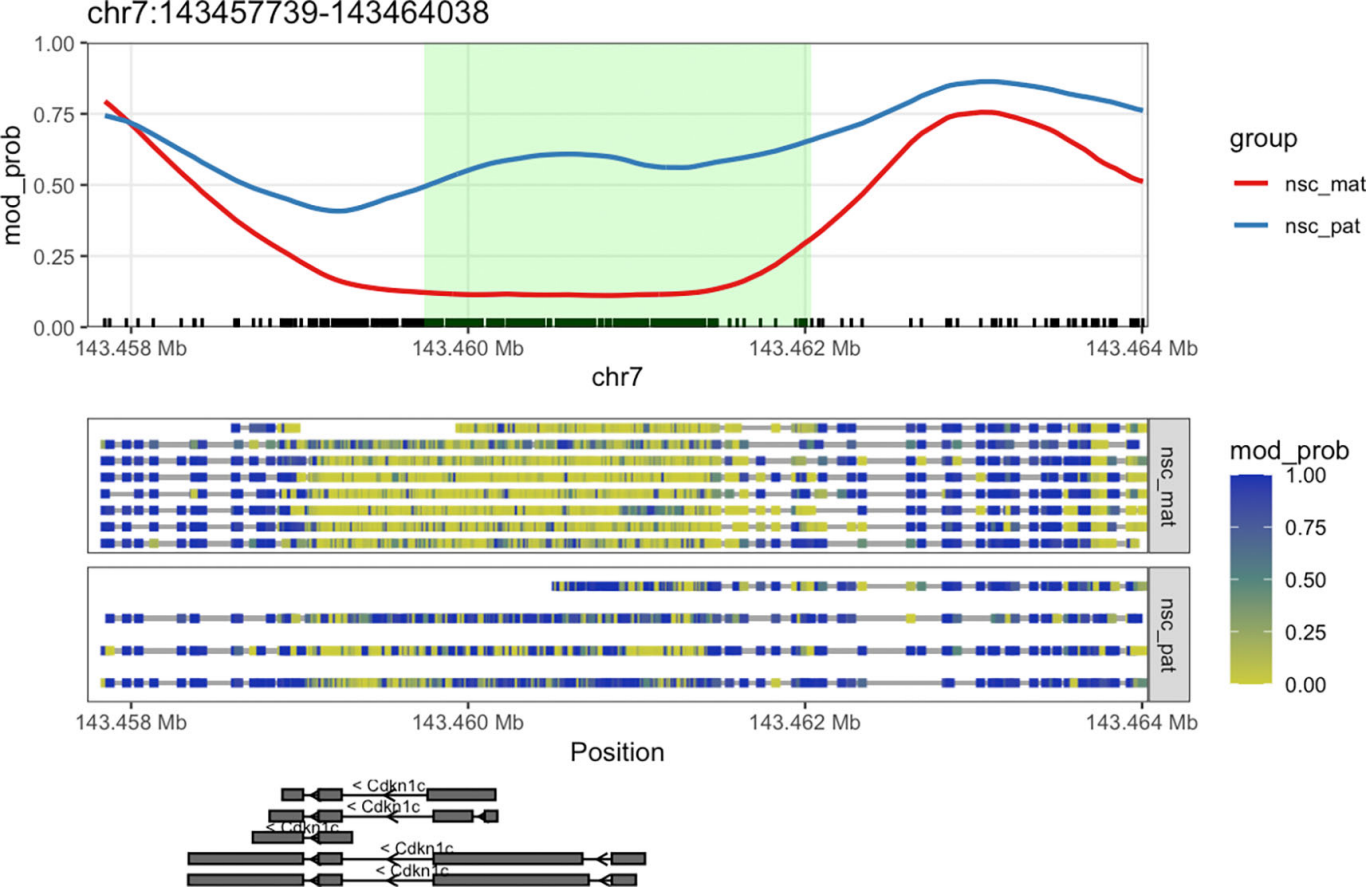
Expanded region around *Cdkn1c.* This plot shows a more expanded region around the gene *Cdkn1c* to show the full DMR.

We can see that the DMR for *Cdkn1c* extends beyond the region that is plotted when we use plot_gene(). We can plot these DMRs using plot_region(), with 2000 bases of flanking sequence added to both sides of the DMR to better visualise the full scope of the differentially methylated region ( Figure 7).

**plot_region**(nmr, "chr7", 143459739-2000, 143462038**+**2000, anno_regions = signif_regions)


We have now successfully used dmrseq to statistically identify differentially methylated regions around known imprinted genes. Additionally, we have used that information to visualise the methylation data of the genes associated with the DMRs, and identify maternal- and paternal-specific methylation of the TSS.

## 3. Summary

In this workflow, we have demonstrated a complete end-to-end pipeline for analysing and visualising methylation data generated by ONT long-read sequencing using NanoMethViz, dmrseq and other Bioconductor packages. We have shown how to preprocess the data, load it into NanoMethViz, visualise the methylation patterns of genes and aggregated features, cluster samples using MDS plots, and identify DMRs between experimental groups. We have also shown how to associate DMRs with genes and visualise the methylation patterns of genes associated with DMRs. While the DMRs identified in this tutorial were all associated with known imprinted genes, this workflow could be used in other experimental contexts to identify candidate genes involved in disease pathogenesis, or discover new gene regulatory elements. Further analysis could include enrichment analysis of the differentially-methylated genes, to bring out pathways that may be up- or downregulated in different processes. Ultimately, this workflow demonstrates a toolkit for the exploration and analysis of methylation data from long-read sequencing, which can be applied to a range of resolution levels and biological contexts. While the workflow demonstrates analysis of 5mC data from the ONT platform, it can also be applied to other long-read DNA methylation data such as that produced by PacBio SMRT sequencing, or other modification types such as 5hmC or 6mA, as well as potentially RNA modifications, provided that data is made available in modBAM format.

## 4. Implementation

This workflow makes use of various open-source R packages available from the R ^18^ and Bioconductor ^19^ projects, with version numbers shown below.

**sessionInfo**()
## R version 4.4.0 (2024-04-24)
## Platform: aarch64-apple-darwin20
## Running under: macOS Sonoma 14.3
##
## Matrix products: default
## BLAS: /Library/Frameworks/R.framework/Versions/4.4-arm64/Resources/lib/libRblas.0.dylib
## LAPACK: /Library/Frameworks/R.framework/Versions/4.4-arm64/Resources/lib/libRlapack.dylib; LAPACK version 3.12.0
##
## locale:
## [1] en_US.UTF-8/en_US.UTF-8/en_US.UTF-8/C/en_US.UTF-8/en_US.UTF-8
##
## time zone: Australia/Melbourne
## tzcode source: internal
##
## attached base packages:
## [1] stats4    stats    graphics    grDevices    datasets    utils    methods
## [8] base
##
## other attached packages:
##  [1] NanoMethViz_3.1.5          ggplot2_3.5.1
##  [3] plyranges_1.24.0           readr_2.1.5
##  [5] dplyr_1.1.4                dmrseq_1.24.1
##  [7] bsseq_1.40.0               SummarizedExperiment_1.34.0
##  [9] Biobase_2.64.0             MatrixGenerics_1.16.0
## [11] matrixStats_1.3.0          GenomicRanges_1.56.1
## [13] GenomeInfoDb_1.40.1        IRanges_2.38.1
## [15] S4Vectors_0.42.1           BiocGenerics_0.50.0
##
## loaded via a namespace (and not attached):
##   [1] splines_4.4.0
##   [2] BiocIO_1.14.0
##   [3] bitops_1.0-8
##   [4] filelock_1.0.3
##   [5] tibble_3.2.1
##   [6] R.oo_1.26.0
##   [7] XML_3.99-0.17
##   [8] lifecycle_1.0.4
##   [9] lattice_0.22-6
##  [10] vroom_1.6.5
##  [11] magrittr_2.0.3
##  [12] limma_3.60.4
##  [13] rmarkdown_2.28
##  [14] yaml_2.3.10
##  [15] doRNG_1.8.6
##  [16] DBI_1.2.3
##  [17] RColorBrewer_1.1-3
##  [18] abind_1.4-5
##  [19] zlibbioc_1.50.0
##  [20] purrr_1.0.2
##  [21] R.utils_2.12.3
##  [22] RCurl_1.98-1.16
##  [23] rappdirs_0.3.3
##  [24] cpp11_0.5.0
##  [25] GenomeInfoDbData_1.2.12
##  [26] irlba_2.3.5.1
##  [27] permute_0.9-7
##  [28] DelayedMatrixStats_1.26.0
##  [29] codetools_0.2-20
##  [30] DelayedArray_0.30.1
##  [31] tidyselect_1.2.1
##  [32] outliers_0.15
##  [33] UCSC.utils_1.0.0
##  [34] farver_2.1.2
##  [35] ScaledMatrix_1.12.0
##  [36] BiocFileCache_2.12.0
##  [37] GenomicAlignments_1.40.0
##  [38] jsonlite_1.8.8
##  [39] annotatr_1.30.0
##  [40] e1071_1.7-14
##  [41] iterators_1.0.14
##  [42] foreach_1.5.2
##  [43] dbscan_1.2-0
##  [44] tools_4.4.0
##  [45] Rcpp_1.0.13
##  [46] glue_1.7.0
##  [47] SparseArray_1.4.8
##  [48] xfun_0.47
##  [49] mgcv_1.9-1
##  [50] HDF5Array_1.32.1
##  [51] withr_3.0.1
##  [52] BiocManager_1.30.25
##  [53] fastmap_1.2.0
##  [54] rhdf5filters_1.16.0
##  [55] fansi_1.0.6
##  [56] digest_0.6.37
##  [57] rsvd_1.0.5
##  [58] R6_2.5.1
##  [59] colorspace_2.1-1
##  [60] Cairo_1.6-2
##  [61] gtools_3.9.5
##  [62] RSQLite_2.3.7
##  [63] R.methodsS3_1.8.2
##  [64] utf8_1.2.4
##  [65] tidyr_1.3.1
##  [66] generics_0.1.3
##  [67] renv_1.0.7
##  [68] data.table_1.16.0
##  [69] rtracklayer_1.64.0
##  [70] class_7.3-22
##  [71] httr_1.4.7
##  [72] S4Arrays_1.4.1
##  [73] org.Mm.eg.db_3.19.1
##  [74] regioneR_1.36.0
##  [75] pkgconfig_2.0.3
##  [76] gtable_0.3.5
##  [77] blob_1.2.4
##  [78] XVector_0.44.0
##  [79] htmltools_0.5.8.1
##  [80] bookdown_0.40
##  [81] scales_1.3.0
##  [82] png_0.1-8
##  [83] knitr_1.48
##  [84] tzdb_0.4.0
##  [85] reshape2_1.4.4
##  [86] rjson_0.2.22
##  [87] nlme_3.1-166
##  [88] curl_5.2.2
##  [89] bumphunter_1.46.0
##  [90] proxy_0.4-27
##  [91] cachem_1.1.0
##  [92] rhdf5_2.48.0
##  [93] stringr_1.5.1
##  [94] BiocVersion_3.19.1
##  [95] parallel_4.4.0
##  [96] vipor_0.4.7
##  [97] AnnotationDbi_1.66.0
##  [98] ggrastr_1.0.2
##  [99] restfulr_0.0.15
## [100] pillar_1.9.0
## [101] grid_4.4.0
## [102] vctrs_0.6.5
## [103] BiocSingular_1.20.0
## [104] dbplyr_2.5.0
## [105] beachmat_2.20.0
## [106] beeswarm_0.4.0
## [107] evaluate_0.24.0
## [108] GenomicFeatures_1.56.0
## [109] cli_3.6.3
## [110] locfit_1.5-9.10
## [111] compiler_4.4.0
## [112] Rsamtools_2.20.0
## [113] rlang_1.1.4
## [114] crayon_1.5.3
## [115] rngtools_1.5.2
## [116] labeling_0.4.3
## [117] plyr_1.8.9
## [118] forcats_1.0.0
## [119] fs_1.6.4
## [120] ggbeeswarm_0.7.2
## [121] stringi_1.8.4
## [122] TxDb.Mmusculus.UCSC.mm10.knownGene_3.10.0
## [123] BiocParallel_1.38.0
## [124] assertthat_0.2.1
## [125] munsell_0.5.1
## [126] Biostrings_2.72.1
## [127] Matrix_1.7-0
## [128] BSgenome_1.72.0
## [129] hms_1.1.3
## [130] patchwork_1.2.0
## [131] sparseMatrixStats_1.16.0
## [132] bit64_4.0.5
## [133] Rhdf5lib_1.26.0
## [134] KEGGREST_1.44.1
## [135] statmod_1.5.0
## [136] AnnotationHub_3.12.0
## [137] memoise_2.0.1
## [138] bit_4.0.5


### 4.1 Operation

Using the provided example data, this workflow can be run with 16GB of RAM and 3GB of disk space on a computer with R v4.4.0, Bioconductor v3.19 and the developmental version of NanoMethViz v3.1.5 installed.

## Author contributions

S.S., L.X. and J.L. developed the workflow, performed data analysis, generated figures and wrote the manuscript and T.C., K.B. and M.E.B. generated data analysed in the workflow. P.F.H., M.E.B., Q.G. and M.E.R. supervised the project and wrote the manuscript. All authors read and approved the final manuscript.

## Data Availability

Data files obtained from Gocuk and Lancaster *et al.* 2024 ^12^ that were used in this workflow are available from: Zenodo: Long-read methylation data analysis with NanoMethViz and Bioconductor, DOI: 10.5281/zenodo.12747550. File: input.tar.gz contains modBAM (aligned to mm10, split into haplotypes and subset to chromosome 7) and sample annotation information. License: CC0.

## Software availability

The LongMod123 workflow package is available at https://github.com/mritchielab/LongMod123 and from Bioconductor.
