# Supplementary material for: A streamlined workflow for long-read DNA methylation analysis with NanoMethViz and Bioconductor
Source: F1000Res. 2025 Feb 24;13:1243. Originally published 2024 Oct 17. [Version 2] doi: 10.12688/f1000research.155204.2 (PMC11907187; doi:10.12688/f1000research.155204.2)
Supplement: Peer review history

# Reviewer report: Reviewer response for version 2

**DOI:** 10.5256/f1000research.177831.r368202

**Published:** 2025-03-13

**Authors:** Tim Triche Jr

**Affiliations:** 1 University of Southern California, Michigan State University, Van Andel Institute, Grand Rapids, USA

The authors have addressed my concerns and those of Dr. Korthauer (while additionally noting that EMseq is largely superfluous for long reads!).

 The Peg3 DMR separation is clearly visible in the DMR plot and, for whatever reason, multidimensional scaling seems to have weighted other features more heavily. Such are the perils of unsupervised nonlinear dimension reduction, of which this is a nice example.

 The dependency of runtime on the various components and inputs is a useful addition when considering what to expect and how to pilot a new analysis.

 The workflow is quite useful and my lab will accordingly be employing it for our work. I have no further reservations about this manuscript and appreciate the authors' efforts improving it.

Are the conclusions about the tool and its performance adequately supported by the findings presented in the article?

Yes

Is the rationale for developing the new software tool clearly explained?

Yes

Is the description of the software tool technically sound?

Yes

Are sufficient details of the code, methods and analysis (if applicable) provided to allow replication of the software development and its use by others?

Yes

Is sufficient information provided to allow interpretation of the expected output datasets and any results generated using the tool?

Yes

Reviewer Expertise:

bioinformatics, epigenetics, loss of imprinting

I confirm that I have read this submission and believe that I have an appropriate level of expertise to confirm that it is of an acceptable scientific standard.

# Reviewer report: Reviewer response for version 2

**DOI:** 10.5256/f1000research.177831.r368201

**Published:** 2025-03-11

**Authors:** Keegan Korthauer

**Affiliations:** 1 Department of Statistics, The University of British Columbia Faculty of Science, BC Children's Hospital Research Institute, Vancouver, British Columbia, Canada

The authors have fully addressed all of my comments, and I now approve this version without reservation.

Are the conclusions about the tool and its performance adequately supported by the findings presented in the article?

Yes

Is the rationale for developing the new software tool clearly explained?

Partly

Is the description of the software tool technically sound?

Yes

Are sufficient details of the code, methods and analysis (if applicable) provided to allow replication of the software development and its use by others?

Yes

Is sufficient information provided to allow interpretation of the expected output datasets and any results generated using the tool?

Yes

Reviewer Expertise:

Statistical genomics

I confirm that I have read this submission and believe that I have an appropriate level of expertise to confirm that it is of an acceptable scientific standard.

# Reviewer report: Reviewer response for version 1

**DOI:** 10.5256/f1000research.170344.r338943

**Published:** 2025-01-09

**Authors:** Tim Triche Jr

**Affiliations:** 1 University of Southern California, Michigan State University, Van Andel Institute, Grand Rapids, USA

The paper and workflow is excellent

 Contrasting Figure 3 and Figure 5, we see that parent of origin (allelic) methylation analysis is critically important for imprinted regions like PEG3.  HOWEVER, the authors state that we can see a clear separation for all three replicates along allelic axes.  This does not actually appear to be the case -- nsc_3 maternal and paternal are nearly indistinguishable.  Searching through the text provides no further remarks on why this is so.  For the sake of clarity, it would be highly desirable to discuss the result and its interpretation. 

 dmrseq is great, but it can take a while.  It would be ideal to provide users with some idea of what to expect in terms of timing, e.g. by using system.time() for steps that are compute intensive. 

 BSseq conversion is an issue which deserves a separate article/workflow/discussion on how it could be optimized. Warning the reader is appreciated!

 With minor edits this is a superb manuscript.

Are the conclusions about the tool and its performance adequately supported by the findings presented in the article?

Yes

Is the rationale for developing the new software tool clearly explained?

Yes

Is the description of the software tool technically sound?

Yes

Are sufficient details of the code, methods and analysis (if applicable) provided to allow replication of the software development and its use by others?

Yes

Is sufficient information provided to allow interpretation of the expected output datasets and any results generated using the tool?

Yes

Reviewer Expertise:

bioinformatics, epigenetics, loss of imprinting

I confirm that I have read this submission and believe that I have an appropriate level of expertise to confirm that it is of an acceptable scientific standard, however I have significant reservations, as outlined above.

# Response

**Published:** 2025-02-04

**Authors:** Matthew Ritchie

**Affiliations:** The Walter and Eliza Hall Institute of Medical Research, Australia

We thank the reviewer for their encouraging feedback.

 We have added new text to clarify the interpretation of the MDS plot:

 “Sample 3 shows only a small difference between the haplotypes, indicating that both its alleles have very similar methylation patterns. Given the level of separation of the groups relative to the similarity of alleles on sample 3, we expect to find some but not many differentially methylated regions between the maternal and paternal groups.”

 We have checked that the similarity isn’t due to library size artifacts and that there is no larger separation present in the 3rd or 4th dimensions of the MDS. We investigated and found that at least in Peg3 the magnitude of difference between the alleles for that sample is equal to other samples, further investigation is beyond the scope of this workflow demonstration.

 We have also added more resource usage information into the “Operation” section to help readers determine the amount of resources and time they might need:

 “On a M3 Macbook Pro with 36GB of RAM, the workflow without pre-generated data takes approximately 8 minutes to run. The time consuming steps are the conversion of the modBAM to tabix-tsv (~80s), the conversion from tabix-tsv to BSseq (~40s) and running dmrseq (~140s). Other time-intensive steps include the aggregate plots over 200 genes (~10s) and over CpG islands (~25s). The input data to this workflow is 1.5GB of modBAM files while in the full dataset there is 60GB of modBAM generated from 3 PromethION flowcells. Run times for conversion functions are expected scale linearly with the size of the input data, run time for dmrseq is expected to scale linearly with the number of genomic sites to be tested, and run time for aggregate plots is expected to scale linearly with the number and average width of the features to be aggregated.”

# Reviewer report: Reviewer response for version 1

**DOI:** 10.5256/f1000research.170344.r338940

**Published:** 2024-12-20

**Authors:** Keegan Korthauer

**Affiliations:** 1 Department of Statistics, The University of British Columbia Faculty of Science, BC Children's Hospital Research Institute, Vancouver, British Columbia, Canada

This article details a workflow for analyzing long-read DNA methylation analysis using the Bioconductor package NanoMethViz and additional Bioconductor packages. The article illustrates the main functionality of this software and companion tools using a small subset of a published ONT study of murine imprinting, including: data import, plotting modification probabilities in local annotated regions, summarizing methylation proportions across genomic region sets, and converting to methylated and unmethylated read counts to take advantage of other Bioconductor tools to construct multidimensional scaling plots and visualize differentially methylated regions.

 The workflow centers on the NanoMethViz software, which is has already been detailed in a PLOS Computational Biology article from 2021 ^1^. However, this workflow provides advances in (1) providing a detailed vignette using a real-world example, and (2) illustrates several extensions that allow for seamless integration of complementary tools for analysis of DNA methylation data. I feel the article would be strengthened by explicitly highlighting the advances made to the software since its release (e.g. supporting updated file types, adding conversion functions), as this type of work is too often devalued even though it is just as important to scientific progress as developing brand new tools. I have also detailed minor comments below.

 Minor comments:

- The NanoMethViz package and workflow take as input the modBAM files currently output by ONT long-read sequencing processing software Dorado. It may be helpful to the reader to indicate whether other software currently output this file format (including ONT tools as well as PacBio tools). Does NanoMethViz also provide functionality for output of any other format to facilitate reanalysis of prior studies or integrative analysis? Or are there tools to convert alternate/legacy file formats to modBAM? Although not necessary to offer this functionality, it may be helpful to the reader to know whether this is possible.
- In the overview of DNA methylation measurement technologies in the introduction, when discussing the limitations of bisulfite sequencing, Enzymatic Methyl sequencing (EM-seq) should also be mentioned as alternative short-read sequencing approach.
- Suggest to write out the default y-axis label in plots like the top panel of Figure 2 as “Modification probability” or “Mod probability” for clarity.
- Please clarify the meaning of the thin grey bar in middle panel of Figure 2 – presumably this delineates the existence of a read, but either no CpG present or no methylation probability estimate?
- It is not clear what the tiny pink and blue shapes represent in bottom panel of Figure 2.
- The text references a “Figure C” – is this referring to Figure 1C?
- The utility of the tabix file was not immediately clear to me on first read of the first paragraph of section 2.3 – the addition of a sentence that explicitly states that the tabix file contains the read-level modification probabilities across all sites for all samples (or modified to correct my understanding as necessary) may be helpful.
- It’s not clear how the methylation proportions as displayed in Figure 3 are calculated. Is this the proportion of samples in each group that had an average modification probability of 0.5 or higher at that CpG, averaged over all samples?
- Figure 3 is interpreted as “In this plot we can see that active genes on chromosome 7 tend to be demethylated near the TSS region and hypermethylated throughout the gene body.” However, I understand it to be a plot of 200 randomly selected genes. As a result, I expect that some of the 200 genes are active but some are not.  
- What assumptions are made when converting a NanoMethResult object to a BSseq object (e.g. methylated if greater than 0.5 probability)? Can this be changed by the user?
- The article mentions alternatives to dmrseq that, like dmrseq, were also developed for short-read sequencing data. However, there are alternatives for DMR detection that were developed specifically for ONT long-read sequencing (e.g. Snajder et al 2023 ^2^) that would be helpful to mention.

Are the conclusions about the tool and its performance adequately supported by the findings presented in the article?

Yes

Is the rationale for developing the new software tool clearly explained?

Partly

Is the description of the software tool technically sound?

Yes

Are sufficient details of the code, methods and analysis (if applicable) provided to allow replication of the software development and its use by others?

Yes

Is sufficient information provided to allow interpretation of the expected output datasets and any results generated using the tool?

Yes

Reviewer Expertise:

Statistical genomics

I confirm that I have read this submission and believe that I have an appropriate level of expertise to confirm that it is of an acceptable scientific standard, however I have significant reservations, as outlined above.

## References

[1] : NanoMethViz: An R/Bioconductor package for visualizing long-read methylation data. *PLoS Comput Biol* .2021;17(10) : 10.1371/journal.pcbi.1009524 e1009524 10.1371/journal.pcbi.1009524 34695109 PMC8568149

[2] : pycoMeth: a toolbox for differential methylation testing from Nanopore methylation calls. *Genome Biol* .2023;24(1) : 10.1186/s13059-023-02917-w 83 10.1186/s13059-023-02917-w 37081487 PMC10120131

# Response

**Published:** 2025-02-04

**Authors:** Matthew Ritchie

**Affiliations:** The Walter and Eliza Hall Institute of Medical Research, Australia

We thank the reviewer for their detailed feedback. We have responded to their comments in point-by-point form below. 

**Comment:** I feel the article would be strengthened by explicitly highlighting the advances made to the software since its release (e.g. supporting updated file types, adding conversion functions), as this type of work is too often devalued even though it is just as important to scientific progress as developing brand new tools.

**Response:** We agree and have added the following paragraph at the end of the Introduction to make this more explicit.

 "Since the initial release of `NanoMethViz` in October 2020, the latest version (v3.3.3) has added support for the now *de facto* standard modBam format, as well as the output of the popular software `Modkit` from ONT. Helper functions have been added to retrieve exon level and CpG island annotation for the mouse genome (mm10, GRCm39) and human genomes (hg19, hg38). A heatmap has been incorporated into the main plots to visualise read-wise methylation data and replaces the previous "spaghetti" lines for improved clarity. Older data formats produced by `nanopolish`, `f5c` and `Megalodon` will remain compatible with `NanoMethViz` and the new features are fully backwards compatible with these formats."

**Comment:** The NanoMethViz package and workflow take as input the modBAM files currently output by ONT long-read sequencing processing software Dorado. It may be helpful to the reader to indicate whether other software currently output this file format (including ONT tools as well as PacBio tools). Does NanoMethViz also provide functionality for output of any other format to facilitate reanalysis of prior studies or integrative analysis? Or are there tools to convert alternate/legacy file formats to modBAM? Although not necessary to offer this functionality, it may be helpful to the reader to know whether this is possible.

**Response:** We have added mention of other software that produce modBAM output, including Jasmine for the PacBio platform:

 “Other software that produce modBAM files include `Jasmine` and `ccsmeth` for PacBio HiFi data, as well as older ONT-compatible methylation callers such as `nanopolish` and `megalodon`.“

 Additionally we have added the following regarding older formats:

 “Older data formats produced by `Nanopolish`, `f5c` and `Megalodon` will remain compatible with NanoMethViz and the new features are fully backwards compatible with these formats.”

**Comment:** In the overview of DNA methylation measurement technologies in the introduction, when discussing the limitations of bisulfite sequencing, Enzymatic Methyl sequencing (EM-seq) should also be mentioned as alternative short-read sequencing approach.

**Response:** The introduction has been updated with mention and brief discussion of EM-seq.

**Comment:** Suggest to write out the default y-axis label in plots like the top panel of Figure 2 as “Modification probability” or “Mod probability” for clarity.

**Response:** The latest developmental version of NanoMethViz (v3.3.3) now displays “Mean Modification Probability” as the y-axis for the gene and region plots and the figures have been updated accordingly.

**Comment:** Please clarify the meaning of the thin grey bar in middle panel of Figure 2 – presumably this delineates the existence of a read, but either no CpG present or no methylation probability estimate?

**Response:** The interpretation of the heatmap for read-level data has been clarified in the figure caption for Fig 2 with the additional sentence:

 “Each row of the heatmap shows one or more non-overlapping reads with a grey bar connecting data that came from the same read.”

**Comment:** It is not clear what the tiny pink and blue shapes represent in bottom panel of Figure 2.

**Response: **Thank you for bringing this to our attention. The pink and blue shapes are image artifacts that should not be in the figure - these will be removed in the revised version.

**Comment: **The text references a “Figure C” – is this referring to Figure 1C?

**Response: **Yes - thank you for spotting the missing reference, which has been updated to Figure 1C.

**Comment: **The utility of the tabix file was not immediately clear to me on first read of the first paragraph of section 2.3 – the addition of a sentence that explicitly states that the tabix file contains the read-level modification probabilities across all sites for all samples (or modified to correct my understanding as necessary) may be helpful.

**Response: **Additional sentences have been added to justify why tabix conversion is necessary:

 “This is because modBAM files store read-level data that include irrelevant sequences and alignment information, requiring the traversal of unnecessary data. Each modBAM entry contains information about multiple CpG sites, with subsequent reads potentially overlapping previous ones. In contrast, the tabix-indexed TSV file format represent a single CpG site per row, sorted in genomic order. This sorting ensures that once a genomic region has been processed, it does not need to be revisited. Consequently, this structure enables more efficient parsing and querying strategies for genome-wide analyses, such as streaming aggregated site-level data without retaining sites in memory.”

**Comment:** It’s not clear how the methylation proportions as displayed in Figure 3 are calculated. Is this the proportion of samples in each group that had an average modification probability of 0.5 or higher at that CpG, averaged over all samples?

**Response:** A sentence in the Figure 3 caption has been extended to clarify that the values are averages of methylation proportions in bins along relative position, the preceding text before the figure specifies the 0.5 threshold for binarisation:

 “averaged across all genes in each experimental group in bins along the relative positions to produce a smoothed trendline.”

**Comment:** Figure 3 is interpreted as “In this plot we can see that active genes on chromosome 7 tend to be demethylated near the TSS region and hypermethylated throughout the gene body.” However, I understand it to be a plot of 200 randomly selected genes. As a result, I expect that some of the 200 genes are active but some are not.  

**Response:** Yes, the sample will be a mix of expressed and unexpressed genes in this random selection (if we had matching RNA-seq data, we could have filtered based on expression to select only expressed genes more precisely). The methylation pattern we see here suggests that there is a good number of expressed genes in this random sample and we have updated the text to mention this:

 “In this plot we see that in the sample of 200 genes on chromosome 7 there tends to be demethylated near the TSS region and hypermethylated throughout the gene body. This is consistent with the proposed function of methylation in gene regulation, where methylation in the promoter region of genes is associated with gene silencing, while transcription of active genes is associated with recruitment of methylating mechanisms to the gene body. So the aggregate signal for this random sample of genes suggests some are actively expressed given that the trend follows this expected methylation pattern."

**Comment:** What assumptions are made when converting a NanoMethResult object to a BSseq object (e.g. methylated if greater than 0.5 probability)? Can this be changed by the user?

**Response:** When converting to BSSeq a 0.5 threshold is used. But in the aggregate plots the user is able to set their own threshold for binarisation. An argument to set the threshold for BSSeq object conversion will be added in future versions of the package.

**Comment:** The article mentions alternatives to dmrseq that, like dmrseq, were also developed for short-read sequencing data. However, there are alternatives for DMR detection that were developed specifically for ONT long-read sequencing (e.g. Snajder et al 2023) that would be helpful to mention.

**Response:** We have added mentions of software external to Bioconductor, along with suggestions of how they may be imported into the workflow:

 "Outside of the Bioconductor ecosystem, other popular software exist with DMR detection capabilities, such as `pycoMeth` in Python and `modkit` developed by ONT. Support for the output of external DMR detection software is not provided in `NanoMethViz`, but if they can be converted into a `GenomicRanges` object then they can be visualised using `NanoMethViz` functions."
